# Renal Outcomes and Dosage Considerations Associated with Bisphosphonate Use: A Systematic Review of Randomized Clinical Trials

**DOI:** 10.21203/rs.3.rs-9724722/v1

**Published:** 2026-05-18

**Authors:** Prajith Venkatasubramanian, Reshma Raju, Pratheeksha Sojan Biatris, Sam Johnson Udaya Chander

**Affiliations:** Centre of Excellence for Long-acting Therapeutics, University of Liverpool, United Kingdom.; Department of Health Informatics, Rutgers University, Piscataway, United States of America.; Orange Pharmacy LLC, Sharjah, United Arab Emirates.; Department of Pharmacy Practice, College of Pharmacy, Sri Ramakrishna Institute of Paramedical Sciences, Coimbatore, Tamil Nadu, India

**Keywords:** Bisphosphonates, renal safety, renal toxicity, alendronate, zoledronic acid, ibandronate, risedronate, renal insufficiency, osteoporosis, safety, tolerability, randomized controlled trials

## Abstract

**Background:**

Though bisphosphonates are the gold standard for the treatment of different metabolic bone disorders including osteoporosis for more than five decades, their safety and tolerability in patients with compromised kidney function are not well known. With age-related bone disorders and renal insufficiency becoming more prevalent worldwide, understanding the effect of bisphosphonates on patients with compromised renal function becomes inevitable. This review aims to analyze the clinical data available on safety of bisphosphonates on patients with different levels of renal function.

**Methods:**

A broad search of PubMed, Wiley Online and the Cochrane Central Register of Controlled Trials was conducted to select randomized controlled trials and clinical trials that evaluated the safety and tolerability of bisphosphonate in patients with different levels of renal function between 2000 and 2026.

**Results:**

Out of 1388 titles and abstract reviewed, 17 articles were included in the final analysis using PRISMA 2020 guidelines. Despite one trial showing < 2% increase in serum creatinine from baseline, all the other trials proved that bisphosphonates are safe and well tolerated by the patients with transplanted kidney and with compromised kidney function. Meta analysis of the data provided from eligible clinical trials using RStudio indicated that the proportion of serum creatinine that is increased was < 25% from baseline. Further, random effects model (100% (−0.01)) was performed due to high level of heterogeneity and it indicated that ibandronate 27.93%; alendronate 28.58%; risedronate 27.80% and zoledronate 15.69%. The pooled effect shows that kidney damage by bisphosphonates is not statistically significant.

**Conclusion:**

The evidence from this review suggests that the bisphosphonates are generally well-tolerated with ten trials registering no drug-related withdrawals and other studies showing only very negligible withdrawals due to adverse effects. Even though bisphosphonates are safer to use in compromised renal function i.e., > 30ml/min/1.73m^2^, more cohort studies are required to identify bisphosphonates effects in end stage renal disease (eGFR < 15ml/min/1.73m^2^).

## 1. Introduction

Osteoporosis has become one of the leading global health burdens incurring high costs to the health systems and it is an independent risk factor for fractures in general population with its risk increasing with age with the prevalence steeply rising with increasing lifespan^1^. Bisphosphonates (BPs) are bone-seeking anti-resorptive agents, which are commonly used to treat different forms of osteoporosis. There are many studies supporting the efficacy of bisphosphonates in the treatment of different types of osteoporosis^2–7^. Available in both oral and injectable forms, bisphosphonates are considered as a drug of choice for the prevention of fractures. Bisphosphonates reduce the fracture risk effectively and have increased risk-to-benefit ratio in the treatment of osteoporosis^8^. Although bisphosphonates have long been the gold standard treatment for treating numerous metabolic bone disorders including osteoporosis, myeloma, bone metastasis, Legg-Perthes disease, Anti-cancer, malignant hyperparathyroidism, and other conditions involving bone fragility, they are not usually recommended in patients with compromised kidney function largely because of the drug being excreted through the kidneys, putting more burden on the already failing kidneys^9,10^.

### 1.1 Pharmacokinetics of Bisphosphonates

Bisphosphonates are administered either intravenously or orally. Oral bisphosphonates are absorbed into the bloodstream from the gastrointestinal lumen by two routes: 1. transcellularly, transported through epithelial cells into the blood. 2. intercellularly, where the bisphosphonates gain access to the circulation via the tight junctions between the epithelial cells. The oral bioavailability of BPs is very low, ranging 1–7%. Moreover, oral absorption is impaired in the presence of food and calcium, magnesium, or aluminum containing drinks^10,11^. During absorption, about 40% of BPs gets into the bone and stays there for more than 10 years and are either slowly released back into the systemic circulation or excreted unchanged^12^.

### 1.2 Mechanism of Action of Bisphosphonates

The principal mechanism by which bisphosphonates act is through “osteoclast inhibition.” During osteoclastic bone resorption, bisphosphonates impairs the cell function of osteoclasts by inhibiting their enzyme activity^13^. This makes them a wonderful choice in all bone diseases that are caused by osteoclast activity. In addition, bisphosphonates can reduce the progression of soft tissue calcification and also has the potential to reduce the progression of vascular calcification^14–16^. Since bone remodeling and vascular calcification occur in patients with decreased renal function, there has been an increased interest in administering bisphosphonates as a treatment option to correct the bone and mineral disorders in patients with compromised renal function.

### 1.3 Bisphosphonates and Renal Function

Since BPs are eliminated primarily through kidneys unchanged, it is important to understand the impact of long-term use of BPs on renal function. The safety newsletter of United States Food and Drug Administration (US FDA) reported 24 cases of acute renal failure and renal impairment between April 2007 and February 2009, associated with the use of zoledronic acid in osteoporosis patients^17^. In another report FDA reported 9 cases of renal injury requiring dialysis and 11 cases of fatal acute renal failure between March 2009 and April 2011, associated with the use of zoledronic acid infusion^18^.

As bone and mineral disorders and renal insufficiency become more prevalent with age and BPs being the first line drug for the treatment of bone related disorders, it is critical to understand the impact of BPs on patients with impaired renal function^19^. Since both oral and parenteral BPs carry warnings regarding their use in patients with impaired renal function, understanding its effects on patients with different levels of renal function become inevitable^20,21^. Hence, review aims to examine the up-to-date clinical data available regarding the renal safety bisphosphonates in patients with different levels of renal function and to discuss the utility of bisphosphonates in patients with bone and mineral disorders who have compromised kidney function.

## 2. Methods

### 2.1 Search Strategy

An extensive search was conducted to select randomized controlled trials and clinical trials that evaluated the safety and tolerability of bisphosphonates in patient with different levels of kidney function. We searched PubMed, Wiley Online and Cochrane Central Register of Controlled Trials between the years 2000 and 2026. The keywords used were “bisphosphonates OR alendronate OR risedronate OR ibandronate OR zoledronic acid OR zoledronate OR pamidronate OR etidronate” AND “renal function” OR “kidney function” OR renal OR kidney OR “renal insufficiency” OR “chronic kidney disease” OR CKD OR “renal impairment” OR “renal safety” OR nephrotoxicity OR “acute kidney injury” OR AKI OR creatinine OR “serum creatinine” OR eGFR OR “glomerular filtration rate” AND “randomized controlled trial” OR randomized OR randomised OR “clinical trial” OR trial OR placebo (refer supplementary table 1). Data from randomized controlled trials that study the effects of bisphosphonates were obtained. We included all articles published in English language that reported randomized controlled trials. All trials must have studied the impact of bisphosphonates on renal function. No restrictions were placed on the dose or formulation of the intervention or the biomarkers used to assess the renal function.

### 2.2 Recovery of trials

**Figure 1:**
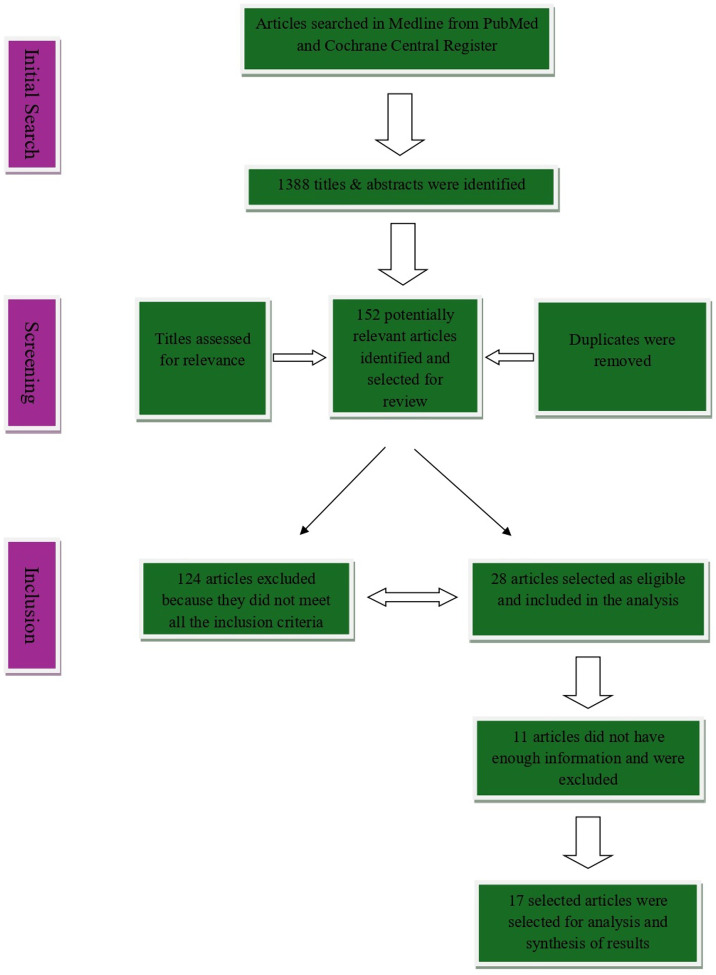
PRISMA Flow Diagram for Study Selection

Our initial search returned 1388 articles (from 30388 records), out of which 152 potentially relevant articles were identified and selected for review. Eligible studies were identified by four authors by screening titles and abstracts by using search keywords as shown in PRISMA table (refer Fig. 1). All trials were then assessed independently by four authors, and relevant studies were selected in accordance with the predefined inclusion criteria. Any disagreement was reviewed and resolved by the fifth independent reviewer. Authors of individual trials were contacted if necessary. After careful review of the abstracts, out of 152 articles, 124 articles that did not satisfy the inclusion criteria were excluded from the analysis. On further scrutiny, out of 124 articles, 11 articles were again excluded owing to lack of critical information. Data from only 17 studies were included in the final review. The flow of article selection process is shown in Fig. 1. The criteria for selection of articles are shown in Table 1.

The eligibility criteria (PICOS) for included RCT was defined

1. *Population*: Adults (> 18 years) with normal or impaired renal function
2. *Intervention*: Bisphosphonates (alendronate, risedronate, ibandronate, zoledronic acid, pamidronate)
3. *Comparator*: Placebo, standard care, or alternative therapy
4. *Outcomes*: Renal safety outcomes including serum creatinine (SCr), eGFR, creatinine clearance, and renal adverse events
5. *Study design*: Randomized controlled trials (RCTs), interventional and registered clinical trials

### 2.3 Data abstraction and study appraisal

We extracted the following general data from each study: country of origin, year of publication, number of randomized patients per each treatment arm, sex ratio, mean age in years, name of the bisphosphonates used in the trial, dose of bisphosphonate used, duration of follow up, and outcomes of the study. The primary outcome of interest was the impact of bisphosphonates on the renal function. Secondary outcomes were the impact of bisphosphonates on improvement in BMD (Bone Mineral Density).

### 2.4 Methodological quality of included trials

The methodological quality of the trials was assessed based on methods of randomization, allocation concealment, blinding, sample size calculation and drop-out rate. For **methods of randomization**, trials were rated as follows: appropriate randomization procedure (A), inappropriate randomization (B), or unclear (C). **Allocation of concealment** was rated as: concealed appropriately (A), not concealed (B), or unclear (C). **Blinding** was rated as: double-blind (A), single blind (B), no blinding (C), or unclear (D). **Sample size calculation** was assessed as: appropriate calculation procedure (A), inappropriate calculation (B), or unclear (C). The drop-out rate **(loss to follow-up**) was assessed as: ≤5.0% (A), 5.1–10.0% (B), 10.1–15.0% (C), > 15.1% (D), or unclear (E)^22^.

## 3. Results

Table 2 summarizes the data from 17 trials selected for final review. The trials were sorted chronologically based on the year the trial was conducted, the year of publication, number of patients per treatment arm, sex ratio, and mean age of the participants.

### 3.1. Patient population:

Between 2003 and 2019, a total of 10766 patients were included. All participants received at least one bisphosphonate along with other medications (if any). The participants were aged 18 years and above and had at least one renal parameter monitored during the study. Participants with both normal and abnormal renal functions were assessed for renal safety with a stipulated period of follow-up according to the study protocol, shown in Table 3.

### 3.2. Primary Endpoint:

Although this review included studies that have analyzed various renal functions, serum creatinine was taken as a primary indicator for renal damage, as it is a standard biochemical measurement of renal function worldwide. Among 17 trials, 8 trials have established the values of SCr while other trials provide eGFR values or other serological tests like creatinine clearance (CrCl), alpha, beta-globulin, TnPO4, PTH and excretion of drug. Table 4 summarizes the effectiveness and tolerability data of bisphosphonates from all 16 studies. Remaining one trial reported outcome measures such as TnPO4, PTH and excretion of drug. Surprisingly, one trial shows that there is a decrease in SCr (−134%) from the baseline. This evidence enhances the safety profile of bisphosphonates in kidney transplantation. Other studies as shown in the table, have a maximum increase of about 7% from the baseline.

### 3.3 Methodological quality of trials

Table 5 summarizes the methodological quality of 17 studies included for final analysis. There was more than 40% loss to follow up in three studies and more than 25% loss to follow up in two studies. It was unclear how randomization was carried out in 11 trials. There were no data available on how allocation concealment was done in any of the studies where blinding was done. 14 trials did not mention how the sample size was arrived at. The methodological quality of trials is shown in Table 5.

### 3.4 Heterogeneity of trials

All the 17 trials included in the final analysis were heterogenous in that they had various inclusion and exclusion criteria and different treatment protocols, which are shown in Table 2 and Table 3.

### 3.5. Meta-analysis of SCr Outcomes from a Subset of RCTs:

**Figure 2:**
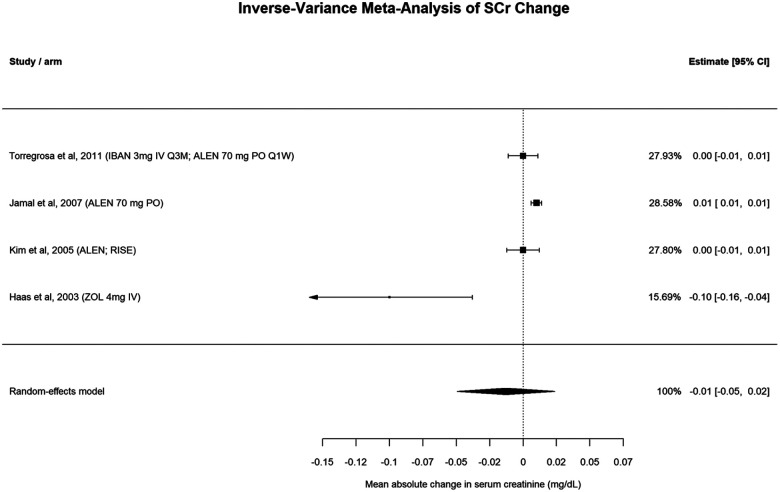
Forest plot of inverse-variance weighted meta-analysis of absolute change in SCr after treatment

Due to substantial heterogeneity in the study design and endpoints, meta-analysis was not feasible across all included trials. Therefore, we included a subset of 3 studies that rigorously reported comparable SCr outcomes and these outcomes were polled using random effects model (as shown in Table 2 & Fig. 2). The 3 selected clinical trials on bisphosphonates which indicate the effect of the drug on the kidney (as shown in Table 6). Data considered for analysis are population with eGFR less than 45 and less than 30 ml/min/1.73m^2^. After performing meta-analysis of the data provided using RStudio (refer Fig. 2), it shows that the proportion of serum creatinine that is increased from baseline is less than 25%, which is not significant according to KDIGO guidelines to cause acute kidney injury. At an outset, the results by Shigematsu *et al*., and Sugimoto *et al*., are loosely based on Hagino *et al*., however, the full results have not been published yet. The results of random effects model (100% (−0.01)) show that ibandronate 27.93%; alendronate 28.58%; risedronate 27.80% and zoledronate 15.69%. These results indicate that bisphosphonate therapy does not significantly alter serum creatinine from baseline.

With this evidence, we postulate that bisphosphonates i.e., Alendronate, risedronate is safer than pamidronate and other bisphosphonates for patients with mild-moderate kidney disease patients (> 30ml/min/1.73m^2^).

## 4. Discussion

Table 3 shows that all the trials included in this analysis have taken SCr levels as their primary outcome, as creatinine is a direct biomarker of renal function. CrCl levels can be calculated from SCr, which was taken as an outcome measure in 9 trials. One trial reported that risedronate caused a significant decrease in eGFR and a significant increase in SCr at 3 and 12 months^23^. Eventhough all the included trials evaluated renal function, only three trials had similar end point (SCr) that can be depicted in a graphical representation (as shown in Fig. 2). Furthermore, the increase in serum creatinine from the baseline, is not significant enough to prove the renal toxicity of bisphosphonates (.i.e. < 25%).

The long-term efficacy of bisphosphonates is rather encouraging. Four trials studied the impact of bisphosphonates on renal function for more than 12 months and these trials reported no detectable change in SCr or only transient changes in renal function, which supports the long-term tolerability of bisphosphonates by the kidneys.^24–27^

Table 2 shows that diverse study population with various comorbid conditions were included in the trials, which ensures that the safety data obtained for this review is unbiased and Table 3 shows that 11 of 16 trials were conducted for at least one year or more, which again reinforces the long-term safety of bisphosphonates on the renal function.

The data from this review suggests that bisphosphonates are generally well-tolerated even in patients with compromised renal function. Ten trials reported no drug-related withdrawals whereas other trials reported either negligible drug-related withdrawal or the data is unclear concerning drug-related withdrawal. Non-serious adverse events resulting from the use of bisphosphonates included mild gastrointestinal distress, eczema, back pain, upper and lower respiratory tract infections, etc. The extent of their safety and efficacy may be different in patients with varying kidney function and it should also be noted that each bisphosphonate is unique in terms of pharmacokinetics. However, based on the results of this review, dosage recommendation of bisphosphonates based on varying levels of kidney function is derived as shown in Table 7.

## Conclusion

Disturbances in serum creatinine and other parameters are hallmark of kidney diseases. Based on the data presented in this review, bisphosphonates maintain normal kidney function in patients with previous history of kidney disease and other comorbid conditions. The review also evidenced that all bisphosphonates except zoledronic acid and risedronate are safe to be used in patients with compromised renal function (> 30ml/min), however evidence of bisphosphonates usage in end stage renal dysfunction is not satisfying and further studies are warranted in cohorts to further discern the tolerability of zoledronic acid, alendronate, pamidronate and risedronate.

## Supplementary Material

This is a list of supplementary files associated with this preprint. Click to download.

- SupplementaryTables.docx

## Figures and Tables

**Table 1:** Characteristics of the Included reported randomised clinical trials

Ref	Country	Year	No. of Randomized Patients Per Each Treatment Arm	Sex Ratio (M/F)	Mean Age in Years
^28^	Brazil	2019	34 (ZA – 17; Control – 17)	10:6; 9:6	43 ±11
^29^	Germany	2015	34 (17 in each arm)	Only women	57.5 ±11.1
^30^	Japan	2014	852 (2.5 mg OD RIS-429; 75 mg OM-423)	8/421; 5/418	67.7 ± 6.0
^23^	Norway	2012	129 (IBN-66; Placebo-63)	48/18; 51/12	51.4 ± 6.5
^31^	Spain	2011	39 (PAM-24; Placebo-15)	14/6; 12/3	48.2 ± 12.3
^32^	USA	2011	801 patients (IBN Inj-268; IBN Inf-264; ALEN-269)	Only women	65.3 ± 4.8
^33^	Australia	2008	24 (Arm 1 = 12; Arm 2 = 12)	07:05; 7:5	59.2 ± 9.2
^24^	Portugal	2008	5035 (ZA – 2521; Placebo – 2514)	Only women	73 ± 5.4
^34^	Spain	2007	84 (Treatment – 39; Control – 45)	20/19; 22/23	56 ± 9.7
^25^	USA	2007	6459 (eGFR < 45 ml/min – 581; eGFR ≥ 45ml/min – 5877)	Only women	72.6 ± 4.4
^35^	Turkey	2007	127 (ALEN – 47; RIS – 44; RAL – 36)	Only women	62.6 ± 7.7
^36^	Korea	2005	44 (Study-22; Control-22)	11/11; 17/5	8.5 ± 2.39
^26^	Belgium	2005	309 patients (Placebo-152; Study-157)	Only women	55.6 ± 12.7
^37^	Spain	2003	26 (Study – 14; Control – 12)	9/5; 7/5	57.3 ± 5.1
^38^	Germany	2002	20 (Placebo – 10; ZA – 10)	4/6; 4/6	52.5 ± 7.8
^27^	USA	2002	58 (Study-29; Control-29)	19/10; 20/9	47.4 ± 2.0
^39^	USA	2000	21 patients (G 1–6; G 2–6; G 3–6; G 4–3)	4/2; 1/5; 6/0; 3/0	59.7 ± 9.2

M/F – Male/Female; OD – Once daily; OM – Once monthly; RIS – risedronate; PAM – pamidronate; IBN – ibandronate; ALEN – alendronate; ZA – Zoledronic acid; RAL – Raloxifene; G - Group

**Table 2:** Change in Serum Creatinine before and after bisphosphonate treatment from baseline

Drug Arm	Baseline SCr(mg/dl)	After Treatment SCr(mg/dl)	Percentage increase in SCr from baseline	Ref
IBAN 6mg; ZOL 4mg	≤ 1.36 mg	Not reported	NA	^29^
IBAN 3mg IVQ3M; ALEN 70 mg PO Q1W	0.82 ± 0.16	0.82	0%	^32^
ALEN 70 mg PO	1.05 ± 0.16	1.06 ± 0.16	0.95%	^25^
ALEN; RISE	ALEN- 0.8 ± 0.2, RISE- 0.8 ± 0.1	ALEN-0.8 ± 0.2, RISE- 0.8 ± 0.2	0%	^36^
PAM	0.527 ± 0.120	0.536 ± 0.122	1.7%	^26^
ZOL 4mg IV	1.5 ± 0.6 mg/dL	1.4 ± 0.1 mg/dL	−6.7%	^38^

SCr- Serum Creatinine; M/F – Male/Female; OD – Once daily; OM – Once monthly; RIS – risedronate; PAM – pamidronate; IBN – ibandronate; ALEN – alendronate; ZA – Zoledronic acid; RAL – Raloxifene; G - Group

**Table 3:** The Methodological Quality of Selected RCTs

Ref	Year	Randomization	Allocation Concealment	Sample Size Calculation	Blinding	Lost to Follow
^29^	2019	A	C	A	A	D
^30^	2015	C	C	C	D	D
^23^	2014	A	C	A	A	D
^31^	2012	A	C	A	A	A
^32^	2011	A	C	C	A	D
^33^	2011	C	B	C	D	B
^24^	2008	C	C	C	D	E
^34^	2008	C	C	C	A	E
^25^	2007	C	C	C	D	A
^35^	2007	C	C	C	D	A
^36^	2007	C	C	C	D	A
^26^	2005	C	C	C	C	A
^37^	2005	C	C	C	A	D
^38^	2003	C	C	C	D	E
^27^	2002	A	C	C	C	D
^39^	2002	A	C	C	C	B
^40^	2000	C	C	C	C	A

**Table 4:** Secondary Analyses of selected RCTs and its significance

Ref	No. of. Patients	Baseline SCr	Follow up SCr	% change from baseline	KDIGO Guidelines (< 25%)
^37^	422	0.76	0.78	2.5%	Not significant
^40,41^	228	0.86 ± 0.12	0.82 ± 0.17	−4.65%	Not significant
^25^	581	1.20 ± 0.2	1.21 ± 0.3	0.8%	Not significant

**Table 5:** PICOS based Study Selection Criteria for Renal Outcomes in Bisphosphonate Research

Population	Inclusion Criteria	Exclusion Criteria
	>18 years, both genders	< 18 years, both genders
Intervention	Bisphosphonates	Regimen without one bisphosphonate at least
Comparator	Any comparator with/without bisphosphonates	NA
Outcomes	Standard Renal parameters.i.e. SCr, eGFR; renal related ADR incidence.	Articles without assessment of renal function viz. SCr, eGFR, etc.
Study Design	Clinical Trial; Randomized Controlled Trial	Observational studies, single case study, reviews, and discussion articles.
Publication	Published in a peer-reviewed academic journals in English language.	Published in any language other than English
Search Database	PubMeD, Cochrane library, Google scholar, Wiley and Online	

**Table 6:** Dosage recommendation of bisphosphonates for clinical practice

Crcl (ml/min)	Zoledronic acid	Alendronate	Risedronate	Pamidronate
50–60	3.5 mg IV	No dosage adjustment	No dosage adjustment	No dosage adjustment
40–49	3.3 mg IV	No dosage adjustment	No dosage adjustment	No dosage adjustment
30–39	3 mg IV	No dosage adjustment	No dosage adjustment	No dosage adjustment
< 30	Contraindicated	Not recommended	Not recommended	Half dose .i.e. 45 mg infuse over 4–6 hours
